# The CX3CL1 intracellular domain exhibits neuroprotection *via* insulin receptor/insulin-like growth factor receptor signaling

**DOI:** 10.1016/j.jbc.2022.102532

**Published:** 2022-09-24

**Authors:** Manoshi Gayen, Marc R. Benoit, Qingyuan Fan, Jacob Hudobenko, Riqiang Yan

**Affiliations:** Department of Neuroscience, University of Connecticut Health, Farmington, Connecticut, USA

**Keywords:** Alzheimer’s disease, neurodegeneration, amyloid toxicity, fractalkine, insulin signaling, forkhead transcription factor, CX3CL1, insulin growth factor, insulin receptor, insulin receptor substrate, AD, Alzheimer's disease, cDNA, complementary DNA, CX3CL1-ICD, intracellular domain of CX3CL1, DIV, days *in vitro*, ER, endoplasmic reticulum, HA, hemagglutinin, IGF, insulin-like growth factor, IGF-1R, insulin-like growth factor 1 receptor, InsRβ, insulin receptor β, IRS, insulin receptor substrate, MAP2, microtubule-associated protein 2, N2A, Neuro-2A, NSC, neural stem cell, pFoxo1, phosphorylated Foxo1, pFoxo3, phosphorylated Foxo3, RRID, Research Resource Identifier, SGZ, subgranular zone, SVZ, subventricular zone, Tg-CX3CL1-ct mice, transgenic mice overexpressing membrane-anchored C-terminal CX3CL1 fragment, TGFβ, transforming growth factor beta

## Abstract

CX3CL1, also known as fractalkine, is best known for its signaling activity through interactions with its cognate receptor CX3CR1. However, its intrinsic function that is independent of interaction with CX3CR1 remains to be fully understood. We demonstrate that the intracellular domain of CX3CL1 (CX3CL1-ICD), generated upon sequential cleavages by α-/β-secretase and γ-secretase, initiates a back signaling activity, which mediates direct signal transmission to gene expression in the nucleus. To study this, we fused a synthetic peptide derived from CX3CL1-ICD, named Tet34, with a 13-amino acid tetanus sequence at the N terminus to facilitate translocation into neuronal cells. We show that treatment of mouse neuroblastoma Neuro-2A cells with Tet34, but not its scrambled control (Tet34s), induced cell proliferation, as manifested by changes in protein levels of transcription factors and progrowth molecules cyclin D1, PCNA, Sox5, and Cdk2. Further biochemical assays reveal elevation of phosphorylated insulin receptor β subunit, insulin-like growth factor-1 receptor β subunit, and insulin receptor substrates as well as activation of proliferation-linked kinase AKT. In addition, transgenic mice overexpressing membrane-anchored C-terminal CX3CL1 also exhibited activation of insulin/insulin-like growth factor-1 receptor signaling. Remarkably, we found that this Tet34 peptide, but not Tet34s, protected against endoplasmic reticulum stress and cellular apoptosis when Neuro-2A cells were challenged with toxic oligomers of β-amyloid peptide or hydrogen peroxide. Taken together, our results suggest that CX3CL1-ICD may have translational potential for neuroprotection in Alzheimer’s disease and for disorders resulting from insulin resistance.

The type-1 transmembrane chemokine CX3CL1, also known as fractalkine, is known to exert its signaling function by binding to its cognate receptor CX3CR1 (1, 2). In the brain, CX3CL1 is largely expressed by neurons, whereas its receptor CX3CR1 is predominantly expressed by microglia (3); this ligand–receptor interaction in the brain triggers neuron–microglia crosstalk by altering neuroinflammatory responses and causing neurotoxic or neuroprotective effects depending on various neurological diseases (4, 5). Recently, we have revealed an intrinsic back-signaling activity of CX3CL1, resulting from its intracellular domain (CX3CL1-ICD), which is generated after sequential cleavage of membrane-bound CX3CL1 by α-, β-, and γ-secretases. Like Notch intracellular domain, CX3CL1-ICD can also translocate into the cell nucleus to alter expression of many genes (6). This signaling event is independent of CX3CL1–CX3CR1 interactions and has its own signaling properties. When transgenic mice overexpress the C terminus of CX3CL1 in neurons (Tg-CX3CL1-ct mice), enhanced neurogenesis in both the subventricular and subgranular zones (SGZs) is observed (7). Importantly, this enhanced neurogenesis mitigates neuronal loss in Alzheimer’s disease (AD) mice such as 5xFAD mice, which exhibit neurodegeneration because of excessive amyloid deposition resulting from overexpressed mutant amyloid precursor protein and presenilin-1 (6, 8), and PS19 mice, which overexpress mutant tau protein and show broad neuronal loss (9, 10). While this observation is intriguing, how C-terminal CX3CL1 exerts its effects on neuroprotection in these two AD mouse models remains to be fully understood. Here, we asked whether a peptide derived from CX3CL1-ICD would be sufficient to enhance signaling functions and to have a translational potential by utilizing cultured cells.

To address these unmet questions, we first designed a synthetic peptide, named Tet34, which was tagged with a 13-amino acid tetanus sequence at the N terminus to facilitate its specific binding to GT1b ganglioside receptors, which are expressed on the surface of neuronal cells (11). We found that this Tet34 peptide was effectively uptaken by neuroblastoma Neuro-2A (N2A) cells and primary hippocampal neurons and that it entered the nucleus in a time-dependent manner. Remarkably, N2A cells treated with Tet34 showed activation of not only the transforming growth factor beta (TGFβ) signaling pathway as described previously but also the insulin receptor β (InsRβ) and insulin-like growth factor receptor 1β signaling pathways.

In addition, we examined downstream signaling molecules in the insulin/insulin-like growth factor 1 (IGF-1) pathways and found significantly elevated phosphorylation of Foxo-3. Foxo proteins, a subgroup of the Forkhead family of transcription factors characterized by a conserved forkhead helix loop DNA-binding domain (FOX), have diverse cellular functions and are known to have roles in stress, aging, apoptosis, and cell-cycle regulation (12, 13, 14). We indeed observed suppression of apoptotic marker proteins as well as upregulation of multiple cellular proliferation markers, consistent with the observed cell proliferation in N2A cells treated with Tet34. The activation of insulin/IGF-1/Foxo signaling was further validated in Tg-CX3CL1-ct/tTA mice, in which transgene was induced in the early adult stage. Thus, our studies demonstrate that the synthetic peptide retains the inherent signaling induction properties of CX3CL1-ICD. This small peptide should be further explored for therapeutic application to counteract neuronal loss in AD and other neurodegenerative diseases.

## Results

### CX3CL1 intracellular peptide translocates to the cell nucleus and induces cell proliferation

**Figure 1:**
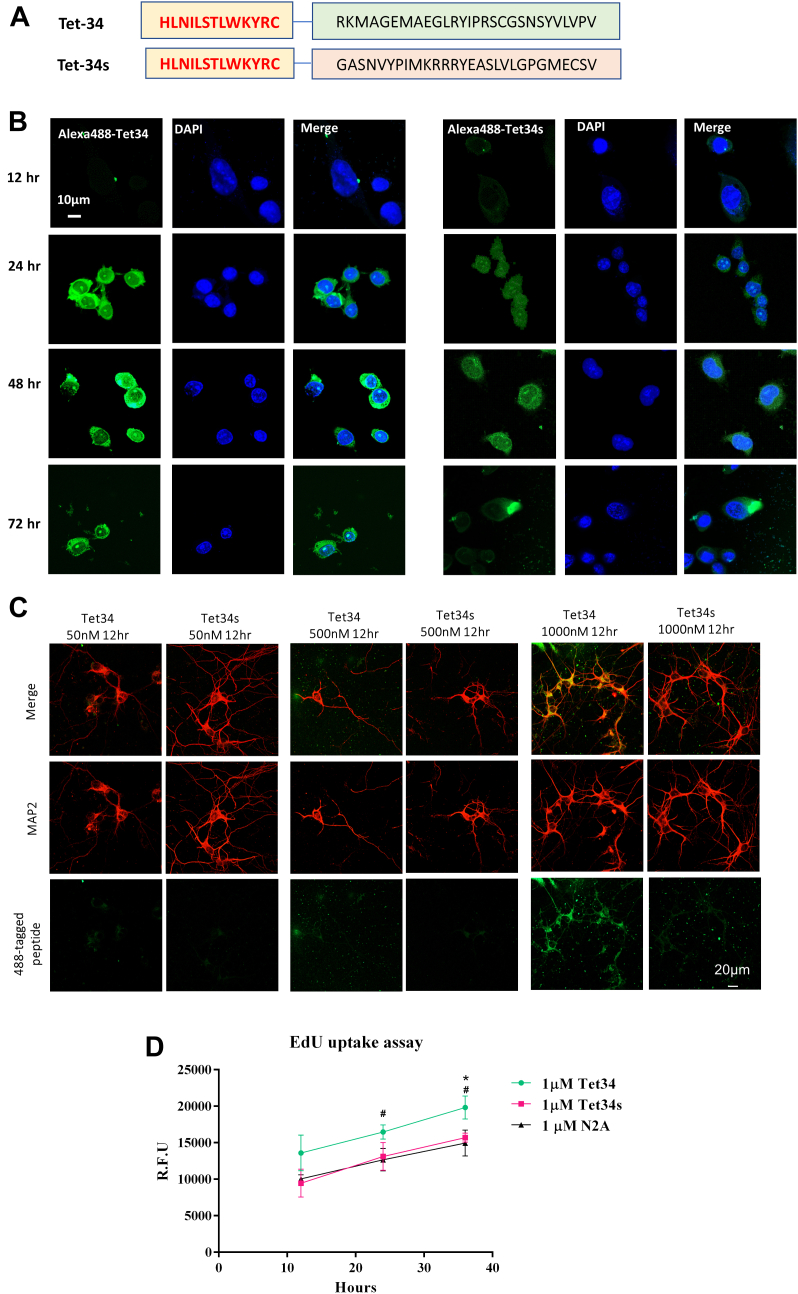
**Designing neuron-specific synthetic CX3CL1-ICD peptides.***A*, schematic representation of the synthetic Tet34, derived from CX3CL1-ICD, and Tet34s peptides. *B*, mouse neuroblastomas-2A (N2A) cells were treated with synthetic Alexa 488-Tet34 peptides for 24 h, which contains an Alexa 488 tag at the terminus of Tet34, followed by fixation at different time points. Alexa 488-Tet34 is retained within cells for up to 72 h, whereas scrambled peptide Tet34s was much less detected in N2A cells. The scale bar represents 10 μm. *C*, peptide Tet34 is selectively uptaken by neurons when the mixed primary brain culture was treated with peptides for 12 h. Scrambled peptide was not readily detected in the mixed primary culture. The scale bar represents 20 μm. *D*, EdU uptake kinetics of N2A, treated with indicated concentrations of Tet34 or Tet34s at 12, 24, and 36 h after treatment, were plotted (N = 3, ∗∗∗*p* < 0.001, Student's *t* test). CX3CL1-ICD, intracellular domain of CX3CL1.

A synthetic peptide was designed from the sequence of CX3CL1-ICD and fused with a 13-amino acid Tet sequence derived from tetanus toxin at the N terminus to improve its specific uptake by neurons and neural stem cells (NSCs; Fig. 1*A*, illustration of peptide sequences). This peptide, named as Tet34, and the control peptide, Tet34s, which has the scramble order of sequence within the CX3CL1-ICD region, were tested for their ability to penetrate into neuronal cells. Since CX3CL1 C-terminal antibody would only recognize Tet34 but not Tet34s, a batch of these peptides were also tagged with Alexa 488 (Alexa488-Tet34 or Alexa488-Tet34s) for monitoring neuronal cell uptake. We showed that Alexa488-Tet34 was more effective in penetration into mouse neuroblastomas N2A cells than Alexa488-Tet34s at concentrations of 125 nM to 200 nM (Fig. 1*B*). During the monition between 12 and 72 h, clear uptake of Alexa488-Tet34 was seen at 24 h and peaked around 48 h.

We also examined uptake of Alexa488-Tet34 in primary neurons cultured from E16.5 mouse hippocampus at varying concentrations and time points. Alexa488-Tet34 and Alexa488-Tet34s were added at concentrations of 50, 500, and 1000 nM at 7 days *in vitro* (DIV) to primary neuron cultures. After treatment for 12 and 24 h, cells were fixed and stained with microtubule-associated protein 2 (MAP2). Alexa488-Tet34 signal was robustly detected in neuronal cell bodies after 12 h at a concentration of 1000 nM (Fig. 1*C*). At the same concentration, Alexa488-Tet34s was not easily detected, suggesting that the sequence of CX3CL1-ICD facilities the uptake of Tet34, although slightly different uptake kinetics between primary hippocampal neurons and N2A cells.

To determine if Alexa488-Tet34 uptake was specific to neurons, we waited until 12 DIV without treatment of AraC to allow proliferation of glial cells. After treatment for 12 h, we began to observe uptake of Alexa488-Tet34 by MAP2-marked neuron, but much less by astrocytes, labeled by GPAP antibody or Iba1-labeled microglia or Olig1-labeled oligodendrocytes (Fig. S1). Again, uptake of Alexa488-Tet34s by neurons or astrocytes was weak. We could not exclude the possibility that a fraction of Alexa488 peptides were phagocytosed and degraded by glial cells. Based on our observations, we conclude that peptide Alexa488-Tet34 is primarily uptaken by neurons. The CX3CL1-ICD sequence appears to facilitate cellular and nuclear uptake when comparing the neuronal uptake by Alexa488-Tet34 and Alexa488-Tet34s.

During the cellular assay, we noted that N2A cells, when treated with Alexa488-Tet34, grew more densely. Therefore, N2A cells were treated with nonfluorescent Tet34 peptides at the concentrations of 1 μM for 12, 24, and 36 h to monitor cell proliferation by EdU incorporation assay. Cells treated with Tet34 had significantly increased EdU incorporation at 24 and 36 h compared with untreated controls or Tet34s-treated cells (Fig. 1*D*), indicating that Tet34 likely has a role in upregulation of cellular proliferation.

### Peptide Tet34 activates the insulin/IGF-1 signaling pathways in cultured cells

To determine how Tet34 upregulates cell proliferation, we first examined whether this fusion Tet34 peptide retains the activity of inducing TGFβ signaling, similar to the expression of CX3CL1-ct in mice (6). We found that treatment of N2A cells with 2 μM of Tet34 for 24 and 48 h significantly increased TGFβ2 and TGFβ3 expression, whereas TGFβ1 levels were elevated only at 48 h, as compared with Tet34s treatment (Fig. S2, *A* and *B*), indicating that Tet34 has a desired signaling activity. Elevated TGFβ2 and TGFβ3 expression induced a significant increase in phosphorylated Smad1 and Smad2 levels at 48 h but not total Smad expression levels (Fig. S2, *A* and *B*). Thus, we demonstrated that fusion of Tet sequence to CX3CL1-ICD retains the activation of the TGFβ/Smad signaling pathway.

**Figure 2:**
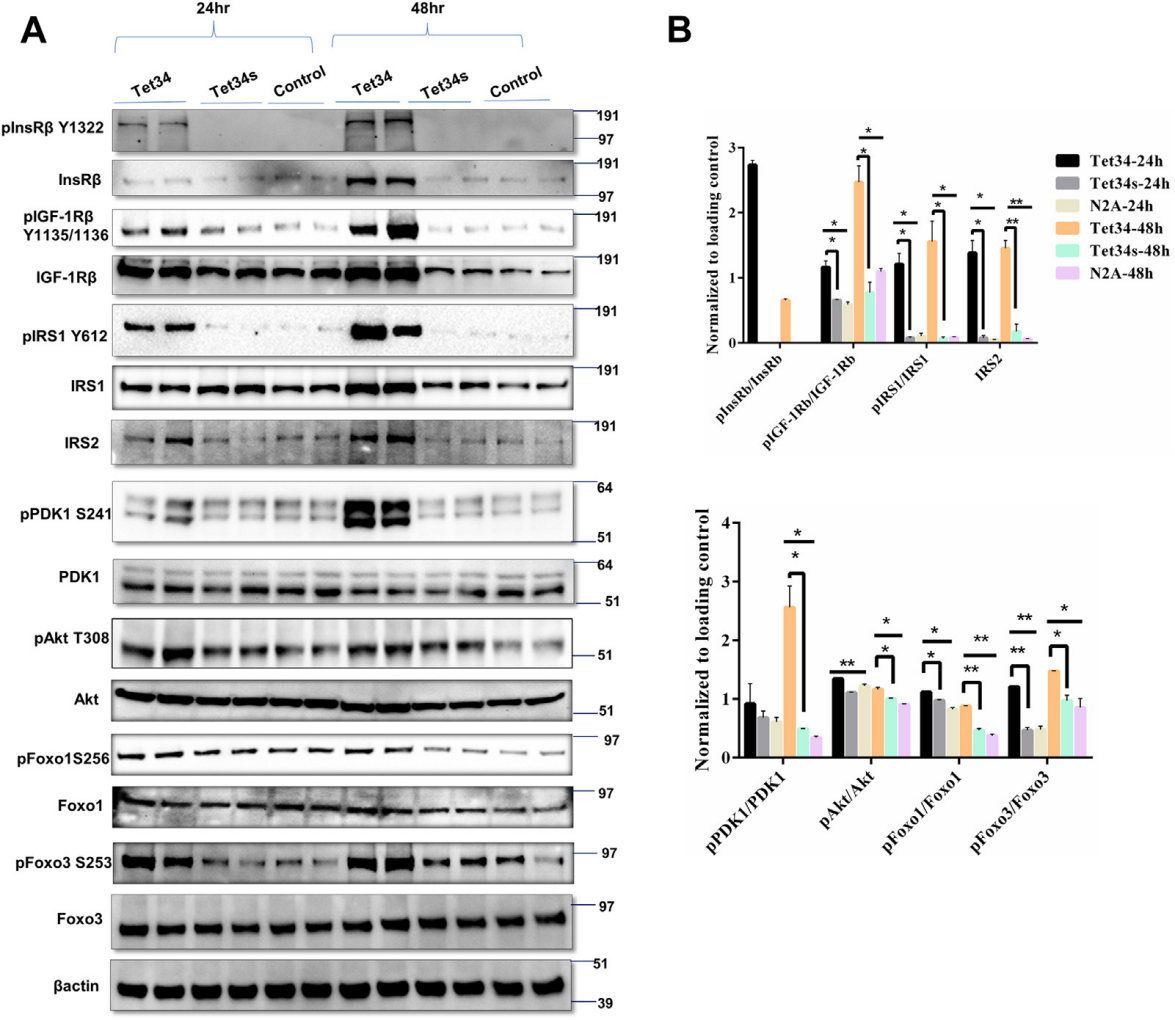
**CX3CL1-ICD peptides induce insulin/IGF-1 signaling pathways.***A*, protein lysates from mouse neuroblastoma-2A (N2A) cells, treated with Tet34 or controls, were examined by the indicated antibodies. *B*, bar graphs compared levels between Tet34 treatment group *versus* Tet34s or Tet34 *versus* control. Tet34 treatment for 24 or 48 h significantly differentially upregulated the phosphorylation levels of key mediators in the insulin/IGF pathway (N = 3 experiments; ∗*p* < 0.05; ∗∗*p* < 0.01; ∗∗∗*p* < 0.001, one-way ANOVA). CX3CL1-ICD, intracellular domain of CX3CL1; IGF, insulin growth factor.

Our further biochemical exploration revealed that Tet34 treatment of N2A cells significantly upregulated expression of InsRβ subunit, which is phosphorylated when activated by insulin (15, 16, 17), when compared with Tet34s or mock treatment (Fig. 2*A*). A corresponding increase in the level of phosphorylated InsRβ was more evident. This increase of phosphorylated InsRβ likely induced activation of its downstream molecules insulin receptor substrate-1 and -2 (IRS1 and IRS2; Fig. 2*A*); the increase was visibly more at the 48 h treatment. Phosphorylated IRS1 was significantly increased and confirmed by quantification (Fig. 2*B*). We also noted an increase in the levels of insulin growth factor 1 receptor (IGF-1R), detected by the antibody specific to the β-subunit (IGF-1Rβ). Levels of pIGF-1Rβ were notably increased as well (Fig. 2, *A* and *B*).

We then further examined downstream molecules and detected activation of PDK1 and Akt, as demonstrated by an increase in their phosphorylated levels but not total PDK1 or Akt (Fig. 2, *A* and *B*). Akt phosphorylation is known to be one of the key regulators of Forkhead transcription factors (Foxos) (13). The activities of Foxos are dependent upon their subcellular localization and phosphorylation status. Nuclear Foxos actively bind to their transcriptional targets, whereas phosphorylated Foxos are shuttled out into cytoplasm, where they undergo either dephosphorylation or degradation (18, 19). Tet34 treatment led to a significant increase in phosphorylation of Foxo3 with no changes in total Foxo3 expression (Fig. 2, *A* and *B*). Phosphorylation of Foxo1 was moderate but significantly increased as well, indicating downregulation of Foxo1 and Foxo3 activity.

We also repeated the same set of experiment by including pharmacological inhibition of Akt activation with compound MK2206 (20), in order to probe the effect of Akt activation on Foxo activity in N2A cells, which were treated with 2 μM of Tet34 or Tet-34s for 24 h. It appeared that inhibition of Akt phosphorylation would diminish phosphorylation of GSK3β and Foxo3 (Fig. S3), indicating a critical role of Akt activity in the Akt/Foxo pathway.

### CX3CL1-ICD enhances insulin/IGF-1 signaling pathways *in vivo*

**Figure 3:**
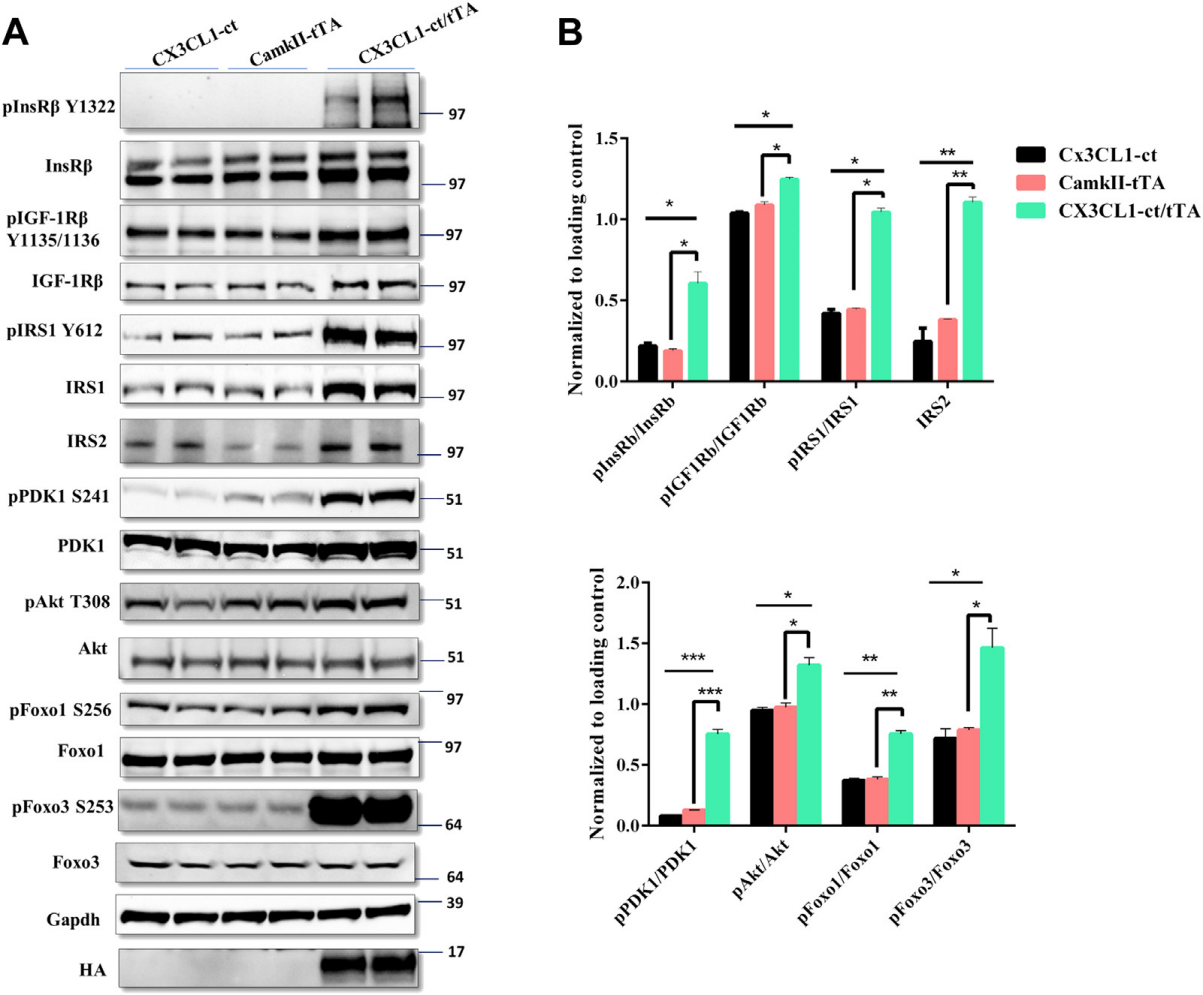
**C-terminal CX3CL1 fragment (CX3CL1-ct) overexpression in transgenic mice induces insulin/IGF-1 signaling pathways.***A*, neuron-specific overexpression of CX3CL1-ct in mice was achieved by breeding CX3CL1-ct mice with CaMKIIa-Tet mice. The transgene was turned on by doxycycline withdrawal at P45. Hippocampal and cortical protein lysates from the indicated genotypes of mice were examined with indicated antibodies by Western blotting. CX3CL1-ct/tTa mice had significantly increased expression of insulin receptor (anti-IGFβ) and insulin growth factor-1 (IGF-1) receptor (IGF-1R; detected by anti-IGF-1Rβ). Downstream molecules insulin substrate 1 (IRS1) and IRS2, Akt, PDK1, and Foxo3 were more obviously activated. *B*, bar graphs show comparative levels normalized to the loading control. N = 3 independent experiments (∗*p* < 0.05; ∗∗*p* < 0.01; ∗∗∗*p* < 0.001, one-way ANOVA). IGF, insulin growth factor; P45, postnatal day 45.

Tg-CX3CL1-ct were generated by utilizing the tetracycline-inducible promoter as described previously (6). This previous study in this model focused on developmental neurogenesis; increased neuron numbers in hippocampal and cortical regions have been demonstrated. To determine whether expression of CX3CL1-ct transgene in adult Tg-CX3CL1-ct/tTA mice would have insulin/IGF-1 signaling functions, we first treated Tg-CX3CL1-ct/tTA mice and their control litters with doxycycline in the drinking water, starting from the mating stage to suppress transgene expression, and doxycycline was removed at the age of postnatal day 45 to turn on CX3CL1-ct expression. After 1 month of induced expression, mice were sacrificed and protein lysates were prepared from hippocampi for Western blot examination. Since CX3CL1-ct has a hemagglutinin (HA) tag on its C terminus, HA expression was observed only in Tg-CX3CL1-ct/tTA mice. When compared with nontransgene-expressing littermates (WT/CX3CL1-ct, CamKII-tTA mice), we found that induced expression of CX3CL1-ct elevated protein levels of both IGF-1Rβ and InsRβ signaling molecules (Fig. 3*A*). Increased phosphorylation of insulin receptor was the most prominent, whereas the increase of pIGF-1Rβ was also visible (Fig. 3*A*). These increases were further confirmed by quantification (Fig. 3*B*).

Their downstream signaling molecules were also examined by the Western blot analysis. As shown in Figure 3*A*, total IRS1 and IRS2 levels were visibly elevated, as was phosphorylated IRS1 levels. While total PDK1 levels were slightly elevated, phosphorylated PDK1 increased significantly (Fig. 3*A*), indicating a strong activation of PDK1 (quantified in Fig. 3*B*). While total Akt levels were not changed, elevation in Akt phosphorylation was significant. Activity of the transcription factors Foxo1 and Foxo3 was both repressed, as the levels of both phosphorylated Foxo1 (pFoxo1) and phosphorylated Foxo3 (pFoxo3) were significantly increased with no changes in total Foxo1 and Foxo3 levels (Fig. 3, *A* and *B*).

Together, these results support that CX3CL1-ICD has a role in the activation of both IGF-1Rβ and InsRβ signaling. Insulin/IGF signaling in brains, specifically in hippocampus, has been shown to regulate spatial learning and memory (21), and this activation may partially explain the improved cognitive functions seen in AD mice (PS19 mice) upon overexpressing CX3CL1-ICD (9).

### CX3CL1-ICD upregulates cellular proliferative markers

**Figure 4:**
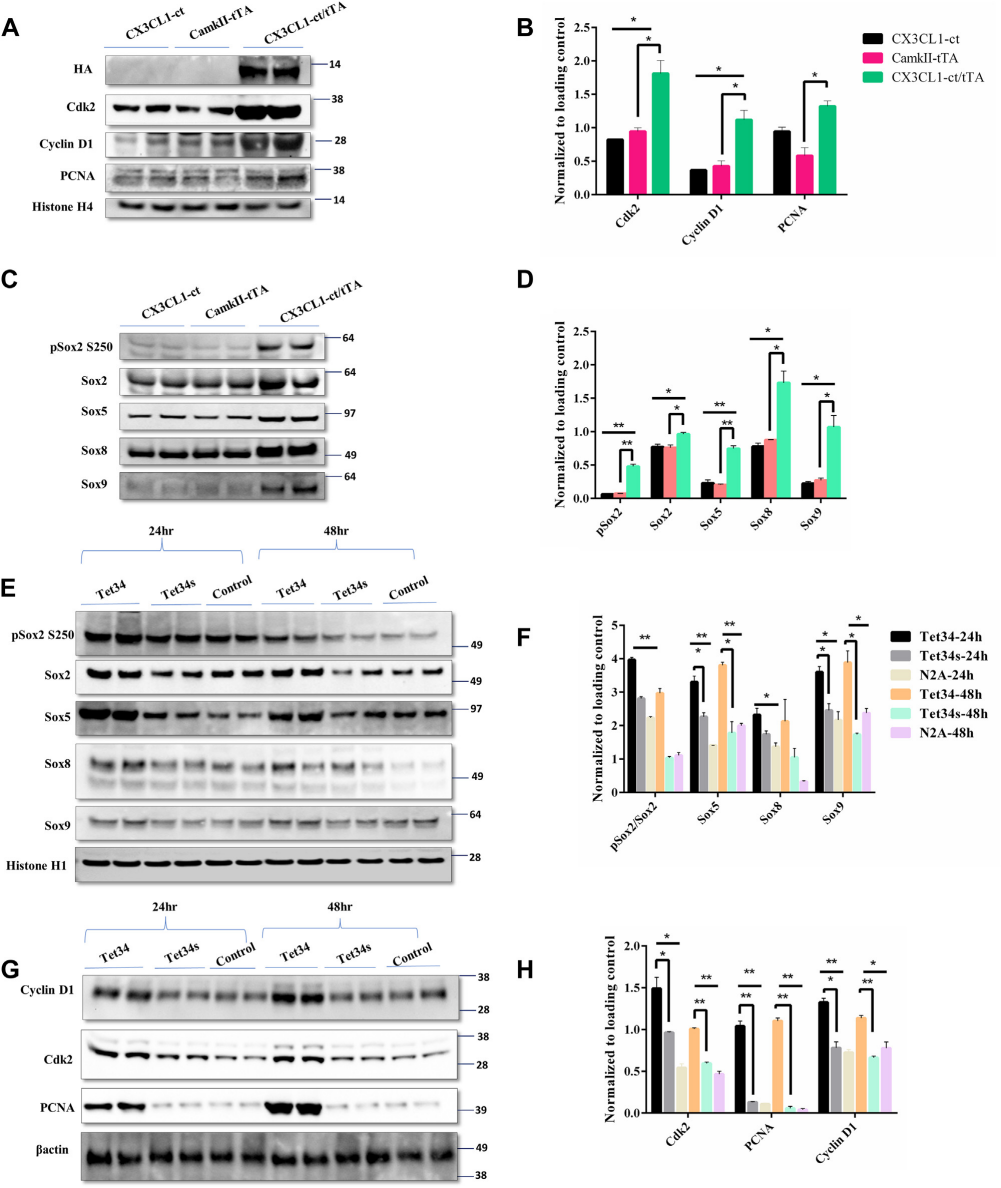
**CX3CL1-ICD induces expression of genes for proliferation.***A*–*D*, significant overexpression of neuronal differentiation markers ASCL1 and NeuroD1 as well as cell cycle regulators, such as cyclin, Cdk, and PCNA, was observed in CX3CL1-ct/tTa mice, using lysates prepared the same as in Figure 3. Sox2, Sox5, Sox8, and Sox9 were also elevated and shown in (*C*). *B* and *D*, bar graphs show comparative protein expression levels normalized to the loading control. N = 3 independent experiments (∗*p* < 0.05; ∗∗*p* < 0.01; ∗∗∗*p* < 0.001, one-way ANOVA). *E* and *G*, the same set of protein levels was compared on the Western blots using protein lysates from N2A cells treated with Tet34 or Tet34s for 24 or 48 h. *F* and *H*, bar graphs show protein expression levels normalized to the loading control. N = 3 independent experiments (∗*p* < 0.05; ∗∗*p* < 0.01; one-way ANOVA). CX3CL1-ICD, intracellular domain of CX3CL1.

Since Foxo proteins are known to regulate cellular proliferation (22, 23), we examined protein levels of transcription factors that control neuronal proliferation in Tg-CX3CL1-ct/tTA mice. We found that proteins important for cell-cycle progression, such as cyclin D1, Cdk2, and PCNA, were significantly elevated (Fig. 4, *A* and *B*). This corroborates our previous findings of Tg-CX3CL1-ct/tTA mice exhibiting enhanced neuronal proliferation and maturation in the SGZ of the dentate gyrus (6, 9).

Our previous bulk RNA-Seq results showed elevation of Sox2 and Sox5, and we indeed observed elevation of these two protein levels including phosphorylated Sox2 in the aforementioned lysates (Fig. 4*C*). Sox5 is a protein known to control cell cycle progression (24), whereas Sox2 activation is known to play a critical role in the maintenance and differentiation of NSCs (25). Sox9 expression was significantly increased, whereas expression of Sox8 was moderately elevated (Fig. 4, *C* and *D*); Sox9 is shown to promote both basal progenitor proliferation and gliogenesis in developing neocortex (26).

In our cellular assays, we showed that the levels of Sox2 and phosphorylated Sox2, Sox5, Sox8, and Sox9 were elevated even after 24 h treatment in N2A cells (Fig. 4, *E* and *F*). Unlike results in Tg-CX3CL1-ct/tTA mice, elevation of PCNA levels was the most obvious, whereas Cdk2, cyclin D1 levels were significantly increased. Together, these results show that Tet34 treatment has a potent signaling activity, which promotes cellular proliferation, in addition to neural differentiation.

### Overexpression of CX3CL1-ICD attenuates neuronal apoptosis in mouse brains

**Figure 5:**
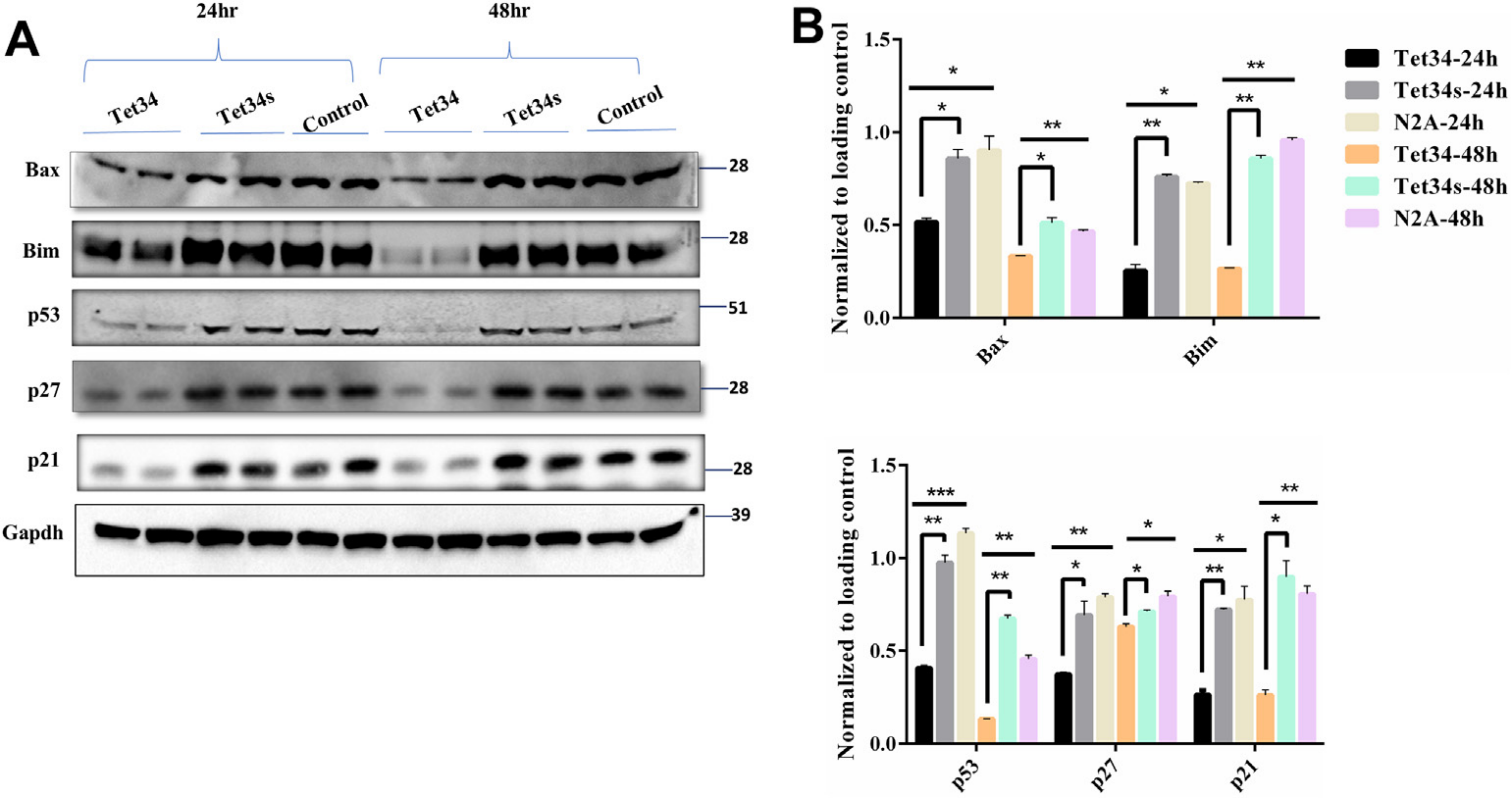
**CX3CL1-ICD peptides repress the apoptotic pathway.***A*, Neuro-2A (N2A) cells treated with Tet34 or Tet34s were prepared for examination by the Western blotting experiment with the indicated antibodies to p53, Bax, p27, and p21. *B*, bar graphs show protein expression levels normalized to the loading control β-actin. N = 3 independent experiments (∗*p* < 0.05; ∗∗*p* < 0.01; one-way ANOVA). CX3CL1-ICD, intracellular domain of CX3CL1.

Foxos are known to be regulators of apoptotic signaling pathways in response to stress (13, 27). N2Acells treated with Tet34 or Tet34s in 2% serum conditions were assayed for the expression of proapoptotic marker proteins, such as p53, Bax, and Bim; these three genes are transcriptionally regulated by Foxos (28). We showed that Tet34 treatment significantly reduced expression of these apoptotic markers in comparison to scrambled Tet34s treatment or mock-treated cells (Fig. 5, *A* and *B*). We showed that while the levels of cyclin-dependent kinase inhibitors p21 and p27 were significantly reduced (Fig. 5, *A* and *B*), consistent with the report that p21 and p27 are transcriptionally regulated by Foxos (28). Significant reduction of these molecules indicates a reduction in cellular apoptosis.

Reduction of neuronal apoptosis is intriguing. SGZ of dentate gyrus and subventricular zone (SVZ) are active neurogenic niches in the murine brain. Majority of the newborn cells in the SGZ and SVZ undergoes apoptosis in different stages of neuronal differentiation and maturation into neurons (29). In line with our previous findings of enhanced neurogenesis (NeuN-positive cells) in the SGZ and SVZ of Tg-CX3CL1-tTA, we found reduction of apoptotic markers in Tg-CX3CL1-tTA mouse hippocampi (Fig. S4). Hence, CX3CL1-ICD may also promote neuronal survival by attenuating apoptosis.

### Tet34 exerts neuroprotection by attenuating Aβ-induced cellular stress and toxicity

**Figure 6:**
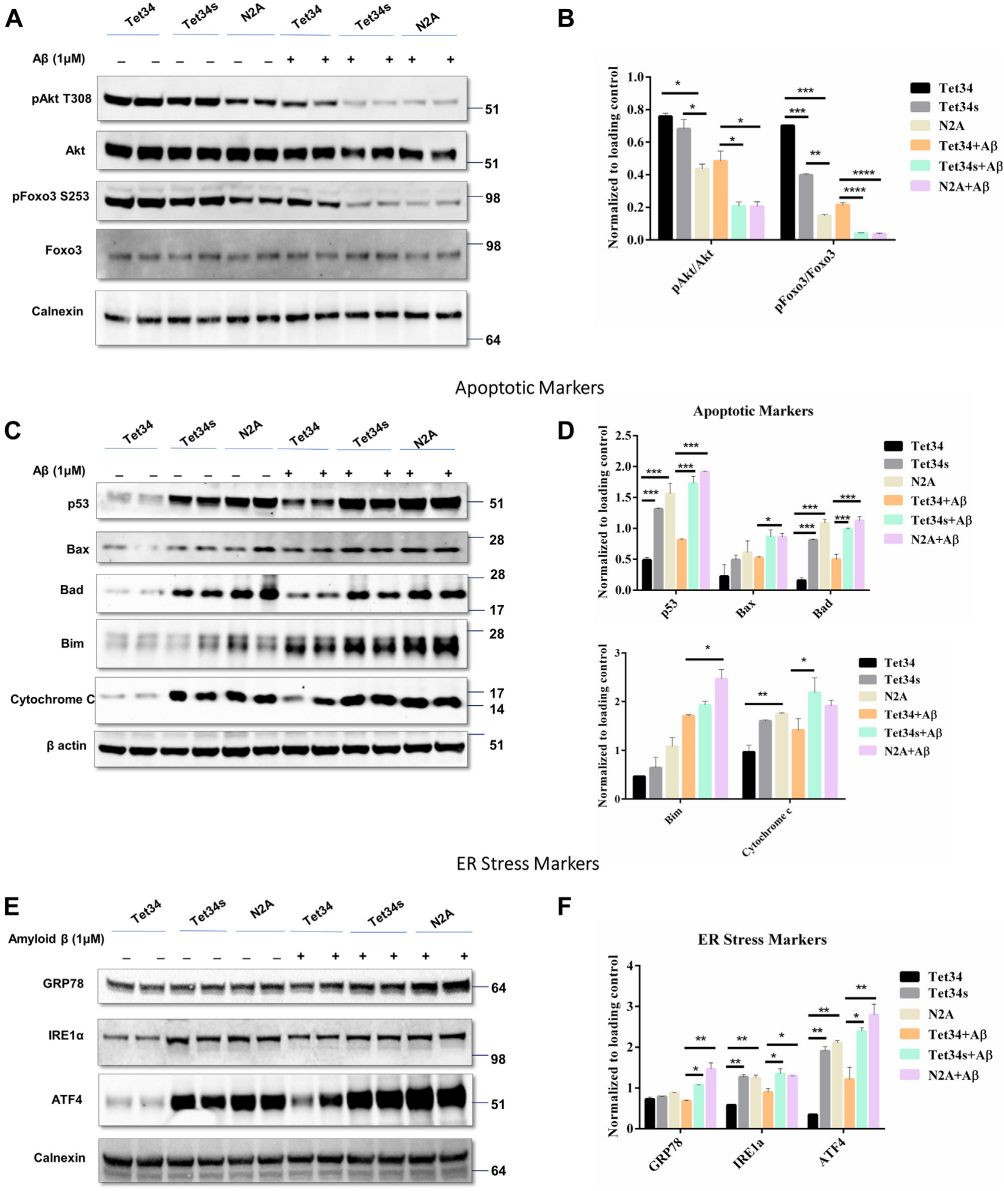
**CX3CL1-ICD peptide confers protection against amyloid beta–induced stress.***A*, Neuro-2A (N2A) cells were treated with 5 μM CX3CL1-ICD peptides for 36 h. A subset of the cells was treated with amyloid beta at 24 h post-treatment with Tet-CX3CL1-ICD peptides for 12 h prior to evaluation of stress-induced markers. *C*, Tet34 significantly attenuated mitochondrial apoptotic markers and (*E*) ameliorated endoplasmic reticulum (ER) stress–induced markers. *B*, *D*, and *F*, bar graphs show protein expression levels normalized to the loading control. n = 3 independent experiments (n = 3 experiments; ∗*p* < 0.05; ∗∗*p* < 0.01; ∗∗∗*p* < 0.001, one-way ANOVA; error bars indicate ±SEM). CX3CL1-ICD, intracellular domain of CX3CL1.

A previous study showed that Aβ treatment of N2A cells induces cellular stress and apoptosis in a dose-dependent manner (30, 31). To determine whether CX3CL1-ICD would exert a neuroprotective effect, we addressed this question in cultured N2A cells. N2A cells were treated with 2 μM of Tet34 and Tet34s for 24 h in 2% serum conditions prior to incubation with 1 μM of oligomeric Aβ_1–42_ (briefed as Aβ thereafter) for 12 h. We showed that Akt protein levels, and its kinase activity, were decreased by Aβ treatment. Correspondingly, less phosphorylation of its substrate, Foxo3, was detected (Fig. 6*A*). N2A cells treated with Tet34 along with Aβ showed clear resilience to Aβ-mediated reduction of pAkt and pFoxo3 levels, which were confirmed by quantification (Fig. 6*B*).

We also noted that Aβ treatment of N2A cells predominantly activated p53, Bad, and Bim, which were elevated when comparing with and without Aβ treatment in N2A cells (Fig. 6, *C* and *D*). Changes in Bax were less obvious. Tet34-treated N2A cells appeared to protect cellular apoptosis as levels of p53, Bad, and Bim were significantly lowered when comparing with Tet34s or mock-treated conditions with or without Aβ challenges. Relatively, Tet34 had less of an attenuating effect on Aβ-mediated Bax activation (*p* = 0.0437) (Fig. 6, *C* and *D*). Reduction of cytochrome *c* release by Tet34 treatment was substantial when comparing Tet34 treated with two control groups (Fig. 6, *C* and *D*); Aβ induced increase in cytochrome *c* release, and this toxic effect was clearly mitigated. These results are in line with the antiapoptotic effects exerted by Tet34. We further confirmed the antiapoptotic effects of Tet34 peptides on N2A cells treated with hydrogen peroxide. Tet34 attenuated the upregulation of various apoptotic markers in both 2% serum conditions as well as in hydrogen peroxide–treated groups (Figs. S5 and S6).

In addition, Aβ is known to induce endoplasmic reticulum stress in neurons (32). Therefore, we asked whether Aβ treatment in N2A cells would also induce endoplasmic reticulum stress and be protected by Tet34 treatment. We found significant increase in the levels of 78-kDa glucose-regulated protein 78 (GRP78), inositol-requiring enzyme 1α, and activating transcription factor 4 proteins in cells treated with Aβ (Fig. 6, *E* and *F*). The expression of these markers was lessened by Tet34 treatment in N2A cells. The 2% serum growth condition by itself also induces low level of stress, and such a low level of stress could also be alleviated by Tet34 treatment. Thus, these results demonstrate that Tet34 exerts protective effects against Aβ-mediated cellular stress and apoptosis.

## Discussion

With the increase in median life expectancy, the onset of age-dependent neurodegenerative diseases has become more prevalent, which has resulted in one of the major social and economic burdens in modern society (33). Thus, finding ways to treat neurodegenerative diseases is an urgent task. In this study, we demonstrate that a short peptide, named Tet34, derived from CX3CL1-ICD, has a therapeutic potential by mitigating neuronal stress and apoptosis.

In this study, we first investigated neuronal delivery of this CX3CL1-ICD-derived short peptide by creating Tet34 peptide and confirmed a relatively higher uptake and retention of Tet34 by N2A cells in comparison to the scrambled Tet34s (Fig. 1*B*). When applied in co-cultures of primary neurons and glia, accumulation of Tet34 in neurons was evident, whereas much less was present in glial cells (Figs. 1*C* and S1). Consistent with our previous findings in transgenic mice expressing C-terminal CX3CL1, Tet34 peptide induced the TGFβ/Smad signaling pathway by significantly upregulated expression of TGFβ2 and TGFβ3, as well as phosphorylation of Smad2 and Smad1 (Fig. S2), indicating that fusion of Tet sequences did not affect the effect of CX3CL1-ICD.

In culture studies, we observed significantly increased cellular proliferation in N2A cells treated with Tet34 (Fig. 1*B*). We previously showed enhanced neurogenesis and significantly more mature neurons in both dentate gurus and cortical regions of Tg-CX3CL1-ct/tTA mice likely because of the activated TGFβ/Smad signaling pathway (6, 9). One intriguing question is whether more mature neurons in the cortical region is due to enhanced adult neurogenesis or decreased apoptosis. It is understood that most of the (50–70%) newborn cells in the SGZ and SVZ undergo programmed cellular death prior to maturation and integration into the neuronal network, and adult neurogenesis in SVZ is less well understood in terms of migration of newborn neurons to the cortical region. With the finding of elevated levels of IGF in our bulk RNA-Seq experiments (6), we asked whether some prosurvival pathways might be playing a crucial role in the survival of the newborn neurons in transgenic mice. In this study, we demonstrated that Tet34 peptides activated insulin/IGF1 signaling pathway. This is the first evidence that CX3CL1-ICD plays a role in mitigating cellular stress and apoptosis through Akt/Foxo pathways.

In both *in vitro* and *in vivo* experiments, we found that peptide Tet34, derived from CX3CL1-ICD, is capable of inducing significant elevation of phosphorylated InsRβ (Y1322) and IGF-1Rβ (Y1134/Y1136) (Figs. 2 and 3). How CX3CL1-ct induces expression of insulin and insulin-like growth factor receptors is intriguing. By analyzing our bulk RNA-Seq data (6), we observed a small increase in gene expression of IGF-1R and IGF binding protein but not insulin receptors. In our RT–PCR experiments, we observed elevation of IGF and IGF-1Rβ mRNA levels (Fig. S7). It is likely that additional post-translational effect may also contribute to the induced expression, and this will be determined in our future studies. Insulin/IGF-1 signaling has been shown to mediate cell-cycle regulation and to modulate cell survival *via* the downstream PDK1/Akt pathway and transcriptional molecules such as Foxos (34). Forkhead transcription factors were identified as Akt substrates, and IGF/Akt signaling regulates the activity of Foxos *via* their phosphorylation followed by nuclear exclusion. In our studies, the increase in Akt phosphorylation correlated with increased phosphorylation of Foxo1 and Foxo3, whereas changes in total Foxo levels were not evident. Phosphorylated Foxos are shuttled out from the nucleus, leading to repression of apoptotic signaling and reversion of Foxo-induced cell cycle control. Blocking Akt phosphorylation at T308 by molecule MK2206 and phosphorylation of GSK3b and Foxo3 were diminished.

Our further investigation into the downstream targets of Foxos uncovered significantly reduced expression of apoptotic marker proteins, such as p53, Bax, and Bim. Cyclin-dependent kinase inhibitors such as p27 and p21 are transcriptional targets of Foxos (35, 36), and we observed marked reductions in these molecules (Fig. 5). Foxos are known to have functional interactions with other cell-proliferative markers such as Cdk2 (37, 38) and cyclin D1 (39). These two molecules were also significantly increased in the Tet34-treated cells. These findings implicate that in addition to TGFβ activation, Tet34, but not Tet34s, promotes cellular survival likely *via* the insulin/IGF-1/Foxo signaling.

Activation of insulin signaling pathway is particularly exciting in the adult because homeostasis of insulin signaling is critical for the modulation of different cellular processes, such as cell survival, autophagy, and cell proliferation (17). In neurons, insulin is implicated in neurite outgrowth, axonal guidance, mitochondrial function, synaptic plasticity, and activity (15, 40, 41, 42). Moreover, the insulin/IGF-1 pathway has also been shown to regulate the exit of neuroblasts from quiescence and promote hippocampal neurogenesis (43, 44). Consistently, Foxos, the downstream transcription factors, are shown to regulate expression of many genes important for adult neurogenesis (45). For example, p21, p27, and p53 are transcriptional targets of Foxos and have vital roles in the maintenance of adult NSC quiescence (35, 46, 47). In Tg-CX3CL1-ct mice, enhanced neurogenesis may potentially be contributed from the increased levels of pFoxo1 and pFoxo3, in addition to the TGFβ/Smad signaling discussed in our previous studies.

Neuronal apoptosis is associated with neurogenesis, physiologic aging, and neurodegenerative conditions (48, 49). Even during neurogenesis, the majority (50–80%) of newborn neurons undergo apoptosis in the process of pruning, establishing neural circuits, and electrical activity (50). IGF-1 has been known to suppress apoptosis (51, 52). Apart from p53, we observed significant attenuation of two other apoptotic markers, Bax and Bim, which are integral members of programmed cell death (apoptosis) and are critical for neurogenesis-associated apoptosis (29, 53, 54). Both Bax and Bim are transcriptional targets of Foxos. We inferred that downregulated Bax and Bim may contribute to reduced neuronal loss in 5xFAD mice expressing CX3CL1-ct [6].

In AD brains, dysregulated insulin/IGF-1 signaling has been found to contribute to neuroinflammation and oxidative stress (55), and insulin signaling dysfunction may underlie disease progression (56, 57). Therapy targeting the insulin-signaling pathway is viewed as a promising approach for treating neurodegenerative diseases. Clinical trials with insulin therapy face challenges because of concerns of off-target effects, limited penetration to the affected areas, and the potential for development of insulin resistance. The ability of CX3CL1-ICD to robustly induce prosurvival and antiapoptotic insulin/Foxo signaling pathways, demonstrated by the activation of their downstream molecules, mitigates the concern of insulin resistance in aging brains. Hence, further exploration of Tet34 for *in vivo* application is imperative and may provide further insights of its dual ability to replenish neuronal loss and confer neuronal protection in various neurodegenerative diseases.

## Experimental procedures

### Synthesis of tagged CX3CL1-ICD peptides and treatments

Peptide named as Tet34 has the sequence of RKMAGEMAEGLRYIPRSCGSNSYVLVPV derived from the CX3CL1 C terminus, whereas the Tet sequence for binding to neuron-specific GT1b receptor HLNILSTLWKYRC was fused to its N terminus. Tet34s has the same GT1b receptor binding sequence, but the remaining sequence has a scrambled order (HLNILSTLWKYRC-GASNVYPIMKRRRYEASLVLGPGMECSV). A batch of peptides were also custom tagged with Alexa Fluor 488 to track the kinetics of cellular and nuclear uptake (Thermo Fisher Scientific). Peptides were custom synthesized (GenScript) and solubilized in 1× PBS to attain a working solution concentration of 2 μg/μl. Multiple aliquots were prepared to avoid repeated freeze–thaw cycles and were stored at −80 °C before use. Monolayers of N2A cells were grown to 70% confluence in either 2-well chamber slides (Thermo Fisher Scientific) or 100 × 20 mm tissue culture–treated dishes (Corning) before treatment with peptides. Cells were treated with 50 nM and 2 μM of peptides in media containing 2% fetal bovine serum for immunostaining and protein analysis, respectively. Postpeptide treatment, cells were incubated at 37 °C, 5% CO_2_ for different time points before analysis.

### Treatment of primary hippocampal cultures with Tet-CX3CL1-ICD peptides

Wildtype C57BL/6J E16.5 mouse embryos were euthanized, and brains were placed in cold Dulbecco’s PBS. The hippocampi were removed, washed with Hank's balanced salt solution, and digested in sterile-filtered papain (1 mg/ml) diluted in Neurobasal media (Gibco; catalog no.: 21103049) for 20 min. Tissues were triturated, and digestion was halted with 10% fetal bovine serum and 1% glutamine in Neurobasal media and filtered through a 70 μM cell strainer. Cells were counted in Trypan blue on a hemocytometer, and 100,000 hippocampal cells were plated onto each chamber slide. Two-well chamber slides were previously coated with poly-d-lysine (0.1 mg/ml) for 24 h at room temperature followed by washing with double-distilled water before plating. Media were exchanged after 24 h with 2% B27-supplement (Thermo Fisher Scientific; catalog no.; 17504044) and 1% glutamine in Neurobasal media. Because of omission of cytarabine (AraC) from culture media, glial cells were allowed to proliferate resulting in a mixed culture. After 12 DIV, cells were treated with 2.5 nM 488-conjugated Tet34 CX3CL1-ICD peptide or Tet34-scrambled for 12 h. Cells were fixed in cold 100% methanol, permeabilized with 0.2% Triton, and blocked in 6% normal goat serum. Slides were incubated in rabbit anti-MAP2 (Millipore; catalog no.: AB5622), rat anti-GFAP (Thermo Fisher Scientific; catalog no.: 13-0300), rabbit anti-Iba1 (Wako; catalog no.: 019-19741), and rabbit anti-Olig2 (Millipore; catalog no.: AB9610) primary antibodies overnight and incubated in Alexa secondary antibodies for 2 h at room temperature. Images were taken on a Zeiss confocal microscope using 63× and 20× objectives. The scale bar represents 20 μm (63×) and 50 μm (20×). Coimmunofluorescence quantification was conducted by measuring 488-pixel intensity within Map2-, Gfap-, and Iba1-positive cell bodies using ImageJ (NIH).

### Cell proliferation assay

N2A cells were grown in 96-well tissue plates and treated with different concentrations of peptides for 24 h. Cell proliferation assays were performed using Click-iT EdU proliferation assay kits (Thermo Fisher Scientific; catalog no.: C10499) according to the manufacturer’s instructions. Briefly, cells were seeded overnight, followed by addition of different concentrations of peptides. The EdU was added to the media after 6 h of peptide treatment. The assay was terminated at 36 h, when Edu incorporation was measured with a microplate reader at 568 nm excitation and 585 nm emission wavelength.

### Tg-CX3CL1-ct transgenic mice

The generation of Tg-CX3CL1-ct mice C-terminal fragment–derived CX3CL1 has been previously described (6). The Tg-CX3CL1-ct pups were genotyped by PCR primers (forward: 5′-CCGATATCTCTGTCGTGGCT-3′ and reverse: 5′-GTTCCTCAGCCTTAGGGGTC-3′). Tg-CX3CL1-ct mice were bred with CaMKIIα-tTA mice (The Jackson Laboratory; catalog no.: 007004) to obtain Tg-CX3CL1-ct/tTA pups. Littermate CX3CL1-ct and CaMKIIα-tTA pups were used as controls. Mice were housed in designated animal rooms at 23 °C on a 12-h light/dark cycle with food and water available *ad libitum*. The doxycycline (Sigma–Aldrich) treatment was at 0.5 mg/ml in drinking water, supplemented with 2% sucrose, and was administered to all animals used in experiment from E0 till postnatal day 45. All experimental protocols were approved by the Institutional Animal Care and Use Committee of the UConn Health Center in compliance with the guidelines established by the Public Health Service Guide for the Care and Use of Laboratory Animals.

### Western blotting and antibodies

Mouse brains were freshly dissected to isolate the hippocampus and cortex, and total proteins were extracted using modified radioimmunoprecipitation assay buffer. Routinely, at least two or three mice from each group were used for Western blot analysis. For *in vitro* studies, proteins were isolated from cells seeded in 6-well tissue culture–treated plates. Equal amounts of protein (50 μg) were resolved on a NuPAGE Bis–Tris gel (Invitrogen) and transferred onto a nitrocellulose membrane (Invitrogen). After protein transfer, the blot was incubated with the indicated antibodies. The sources of antibodies are p-InsRß (Santa Cruz Biotechnology; catalog no.: sc-81501, Research Resource Identifier [RRID]: AB_1125643), InsRß (Cell Signaling Technology; catalog no.: 3020, RRID: AB_2249166), p-IGF-1Rb (Cell Signaling Technology; catalog no.: 3021, RRID: AB_331578), IGF-1Rβ (Cell Signaling Technology; catalog no.: 3027, RRID: AB_2122378), p-IRS1 (Thermo Fisher Scientific; catalog no.: 44816G, RRID: AB_2533768), IRS-1 (Cell Signaling Technology; catalog no.: 3407, RRID: AB_2127860), IRS-2 (Cell Signaling Technology; catalog no.: 4502, RRID: AB_2125774), p-Smad2 (catalog no.: 3104, RRID: AB_390732), Smad2/3 (catalog no.: 8685; RRID: AB_10889933), p-Smad1 (catalog no.: 9553s; RRID: AB_2107775), Smad1 (catalog no.: 6944; RRID: AB_10860070), TGFβ1 (catalog no.: SC146; RRID: AB_632486), TGFβ2 (catalog no.: SC-90; RRID: AB_2303237), TGFβ3 (catalog no.: SC-82; RRID: AB_2202303), pPI3K (Cell Signaling Technology; catalog no.: 17366, RRID: AB_2895293), PI3-kinase p85 (Cell Signaling Technology; catalog no.: 4257, RRID: AB_659889), pGSK3β (Cell Signaling Technology; catalog no.: 9336, RRID: AB_331405), Gsk3β (Cell Signaling Technology; catalog no.: 9315, RRID: AB_490890), PARP (Cell Signaling Technology; catalog no.: 9532, RRID: AB_659884), cleaved caspase 9 (Cell Signaling Technology; catalog no.: 9509, RRID: AB_2073476), pFoxo1 (Cell Signaling Technology; catalog no.: 84192, RRID: AB_2800035), Foxo1 (Cell Signaling Technology; catalog no.: 2880, RRID:AB_2106495), pFoxo3 (Cell Signaling Technology; catalog no.: 13129, RRID: AB_2687495), Foxo3 (Cell Signaling Technology; catalog no.: 12829, RRID: AB_2636990), pPDK1 (Cell Signaling Technology; catalog no.: 9634, RRID: AB_2161307) (Cell Signaling Technology; catalog no.: 3438, RRID: AB_2161134), PDK1 (Cell Signaling Technology; catalog no.: 3062, RRID: AB_2236832), pAKT (Cell Signaling Technology; catalog no.: 13038, RRID: AB_2629447), AKT (Cell Signaling Technology; catalog no.: 4691, RRID: AB_915783), NeuroD1 (Abcam; catalog no.: ab16508, RRID: AB_470254), ASCL1 (Santa Cruz Biotechnology; catalog no.: sc-374104, RRID: AB_10918561), p53 (Santa Cruz Biotechnology; catalog no.: sc-6243, RRID: AB_653753), p21 (Santa Cruz Biotechnology; catalog no.: sc-6246, RRID: AB_628073), p27 (Cell Signaling Technology; catalog no.: 3688, RRID: AB_2077836), Bax (Santa Cruz Biotechnology; catalog no.: sc-493, RRID: AB_2227995), Bim (Cell Signaling Technology; catalog no.: 2933, RRID: AB_1030947), pSox2 (Cell Signaling Technology; catalog no.: 92186, RRID: AB_2800179), Sox2 (Millipore; catalog no.: AB5603, RRID: AB_2286686), Sox5 (Abcam; catalog no.: ab94396, RRID: AB_10859923), Sox8 (Santa Cruz Biotechnology; catalog no.: sc-374446, RRID: AB_10989367), Sox9 (Cell Signaling Technology; catalog no.: 82630, RRID: AB_2665492), HA (Roche; catalog no.: 1867423; RRID: AB_10094468), and CX3CL1 (Santa Cruz, catalog no.: SC7225, RRID: AB_2087136). Horseradish peroxidase–conjugated secondary antibodies were used and visualized using enhanced chemiluminescence (Thermo Fisher Scientific).

### Real-time PCR quantitation

Mouse brains were freshly dissected to isolate the hippocampus and cortex and midbrain. Total RNA was extracted using Trizol (Thermo Fisher Scientific; catalog no.: 15596026) according to the manufacturer’s protocol. About 4 μg of total RNA was used to perform complementary DNA (cDNA) transcription with High capacity cDNA Reverse Transcription kit (Thermo Fisher Scientific; catalog no.: 4368813). About 2 μl of cDNA was used for real-time PCR quantitation of IGF-1Rβ mRNA, IGF1 mRNA, and 18S rRNA with SYBR Green Master Mix (Thermo Fisher Scientific; catalog no.: A25742). The quantitative PCR primers used were IGF-1Rβ (forward: 5′-GTG GGG GCT CGT GTT TCT C-3′ and reverse: 5′-GAT CAC CGT GCA GTT TTC CA-3′), IGF1 (forward: 5′-CCG AGG GGC TTT TAC TTC AAC AA-3′ and reverse 5′-CGG AAG CAA CAC TCA TCC ACA A-3′) and 18S rRNA (forward: 5′-TGTGCCGCTAGAGGTGAAATT-3′ and reverse 5′–TGGCAAATGCTTTCGCTTT-3′).

### Amyloid-β treatment of cultured cells

N2A cell monolayers at 70% confluency were treated with 5 μM Tet-peptides for 24 h. Reconstituted human Aß_1–42_ (Biosource) was added to the cells at a 1 μM concentration and incubated for 12 h. The Aß_1–42_ was reconstituted according to the manufacturer's protocol. Briefly, the lyophilized peptide was dissolved in HPLC water followed by dilution in PBS. The solution was incubated at 37 °C and shaker for 24 h to obtain the neurotoxic form of Aß_1–42_. In our experience, we get ∼70% oligomeric Aß_1–42_ with this protocol. Total cell lysate was collected by radioimmunoprecipitation assay lysis buffer for Western blot evaluation of apoptotic pathways.

### Hydrogen peroxide–induced apoptosis in cultured N2A cells

N2A cell monolayers grown to 70% confluency were treated with 2 μM Tet-peptides for 24 h in 2% serum for signaling induction. The cells were then incubated with 50 μM hydrogen peroxide (Sigma; catalog no.: H1009) for different time points to induce apoptosis. A batch of cells were harvested at 1 h to evaluate apoptosis by Western blots. Flow cytometry using Alexa Fluor 488 Annexin V kit (Thermo Fisher Scientific; catalog no.: V13241) was used to determine changes in the population of cells undergoing apoptosis at different time points of treatment.

### Akt inhibition assay in cultured N2A cells

N2A cell monolayers at 70% confluency were treated with 2 μM Tet-peptides for 24 h in 2% serum for signaling induction. The cells were then incubated with 2 μM MK2206, Akt inhibitor (Selleckchem; catalog no.: S1078) for 3 h prior to total cell lysate collection. Western blot was performed to evaluate the specificity of Tet-peptides in the induction of IGF1Rb/Akt/Foxo signaling pathway.

### Statistical analysis

Quantitative data are presented as mean ± SEM. All experiments were independently repeated at least three times. Statistical analyses were conducted using Prism 6 software (GraphPad Software, Inc). Statistical comparisons between groups were analyzed for significance by one-way ANOVA with Tukey's post hoc test and Student’s *t* test. Significant *p* values are denoted by asterisks in the text and figures (∗*p* < 0.05, ∗∗*p* < 0.01, and ∗∗∗*p* < 0.001). Error bars in each case represent SEM.

## Data availability

All original data presented in the article will be made available for reviews when needed. Research materials will be also made available when it is required.

## Supporting information

This article contains supporting information.

## Conflict of interest

The authors declare that they have no conflicts of interest with the contents of this article.
