# Supplementary material for: The CX3CL1 intracellular domain exhibits neuroprotection via insulin receptor/insulin-like growth factor receptor signaling
Source: J Biol Chem. 2022 Sep 24;298(11):102532. doi: 10.1016/j.jbc.2022.102532 (PMC9626938; doi:10.1016/j.jbc.2022.102532)
Supplement: Supplemental Figures S1–S7 [file mmc1.docx]

**
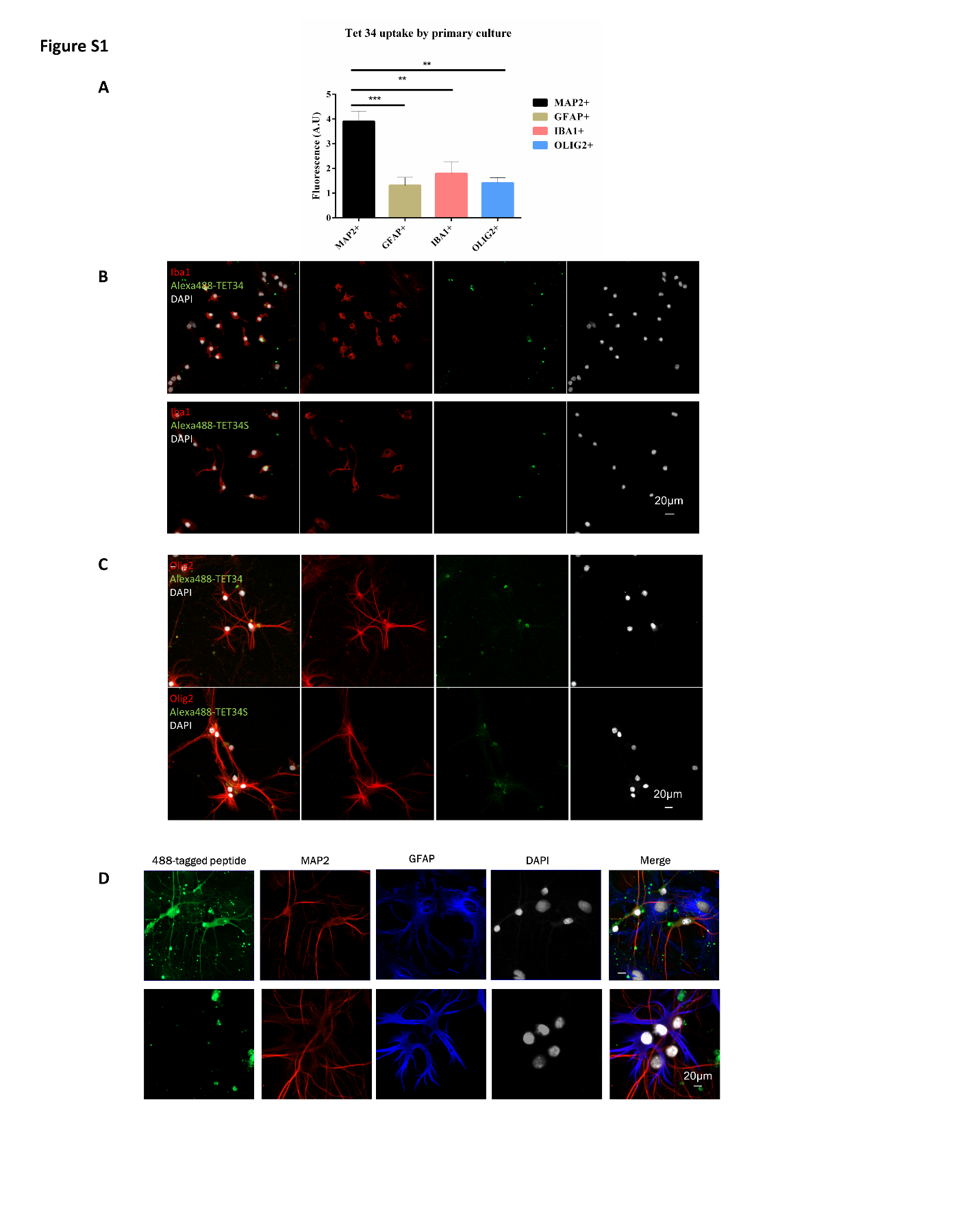
**

**Supplemental Figure S1**: Peptide Alexa488-Tet34 is preferentially uptaken by Map2-marked neurons. Primary mixed brain co-cultures were treated with Alexa488-Tet34 or Alexa488-Tet34s peptides for 12 hrs. Cells were marked by Map2 for neurons, Iba1 for microglia and Olig2 for oligodendrocytes. Images were taken on a Zeiss confocal microscope using 63X or 20X objectives. Scale bar = 20um (63X) and 50um (20X). Co-immunofluorescence quantification was conducted by measuring 488-pixel intensity within Map2-, Gfap-, and Iba1-positive cell bodies using ImageJ. N = 61 neurons, 56 astrocytes, 42 microglia and 19 oligodendrocytes (***P < 0.001, comparing only to neurons; student’s t test). (A) quantification showed intensity co-stained with Alexa488-Tet34 with Map2-marked neurons, or Gfap-marked astrocytes or Iba1-marked microglia. (B-D) Confocal staining of mixed cultures with indicated antibodies for microglia (B), oligodendrocytes (C), neurons and astrocytes (D). Scale bar is 50 µm (B, C) and 20 μm (D).


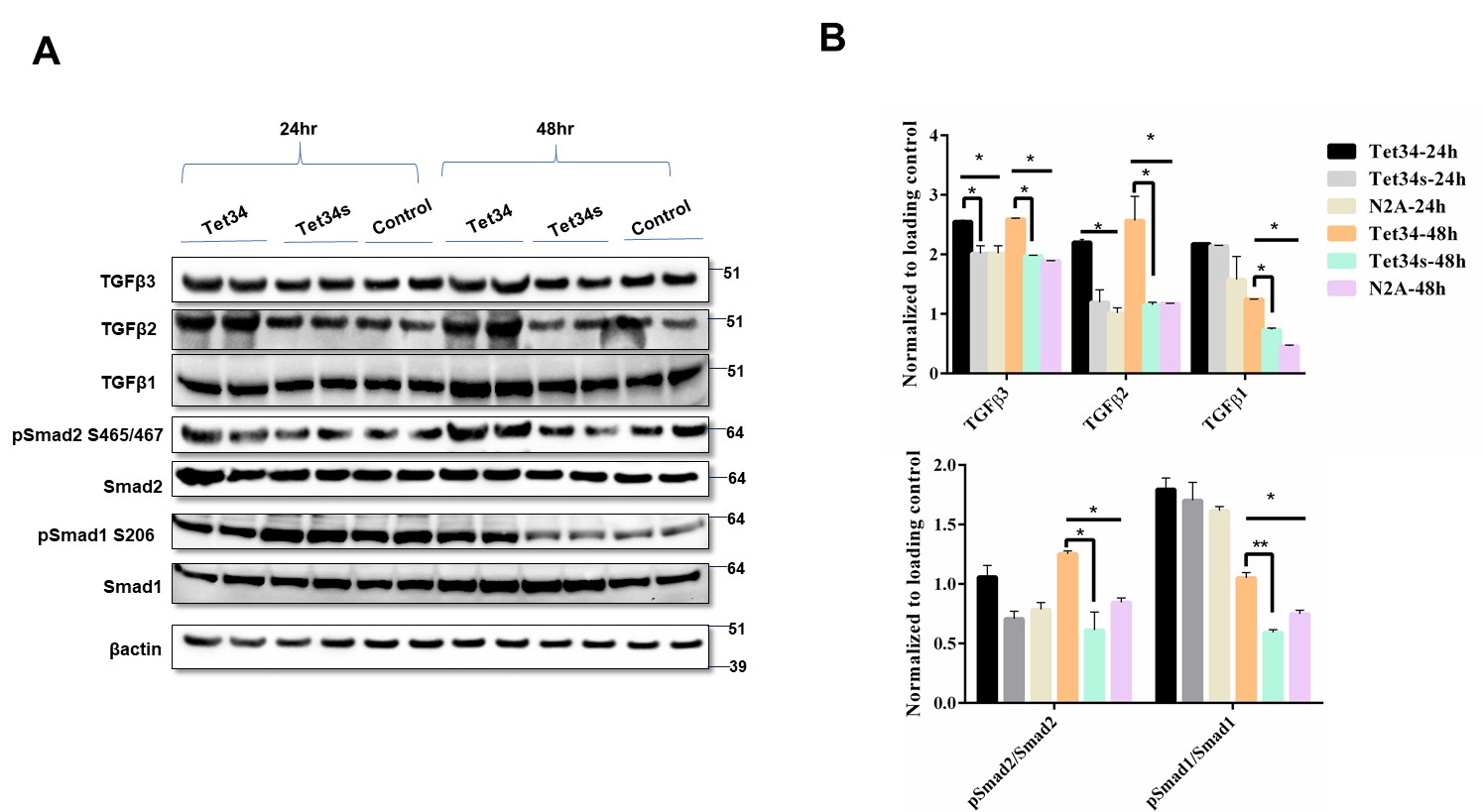


**Supplemental Figure S2**: (**A**) The CX3CL1-ICD-derved Tet34 peptide induce the TGFβ/Smad signaling pathway in N2A cells at 24 hrs and 48 hrs post-treatment in N2A cells. (**B**) Bar graphs show that Tet34 significantly increased TGFβ expression and phosphorylated Smad levels at 48 hrs post-treatment. (N=3 experiments; *P<0.05; **P<0.01; ***P<0.001, one-way ANOVA).

**
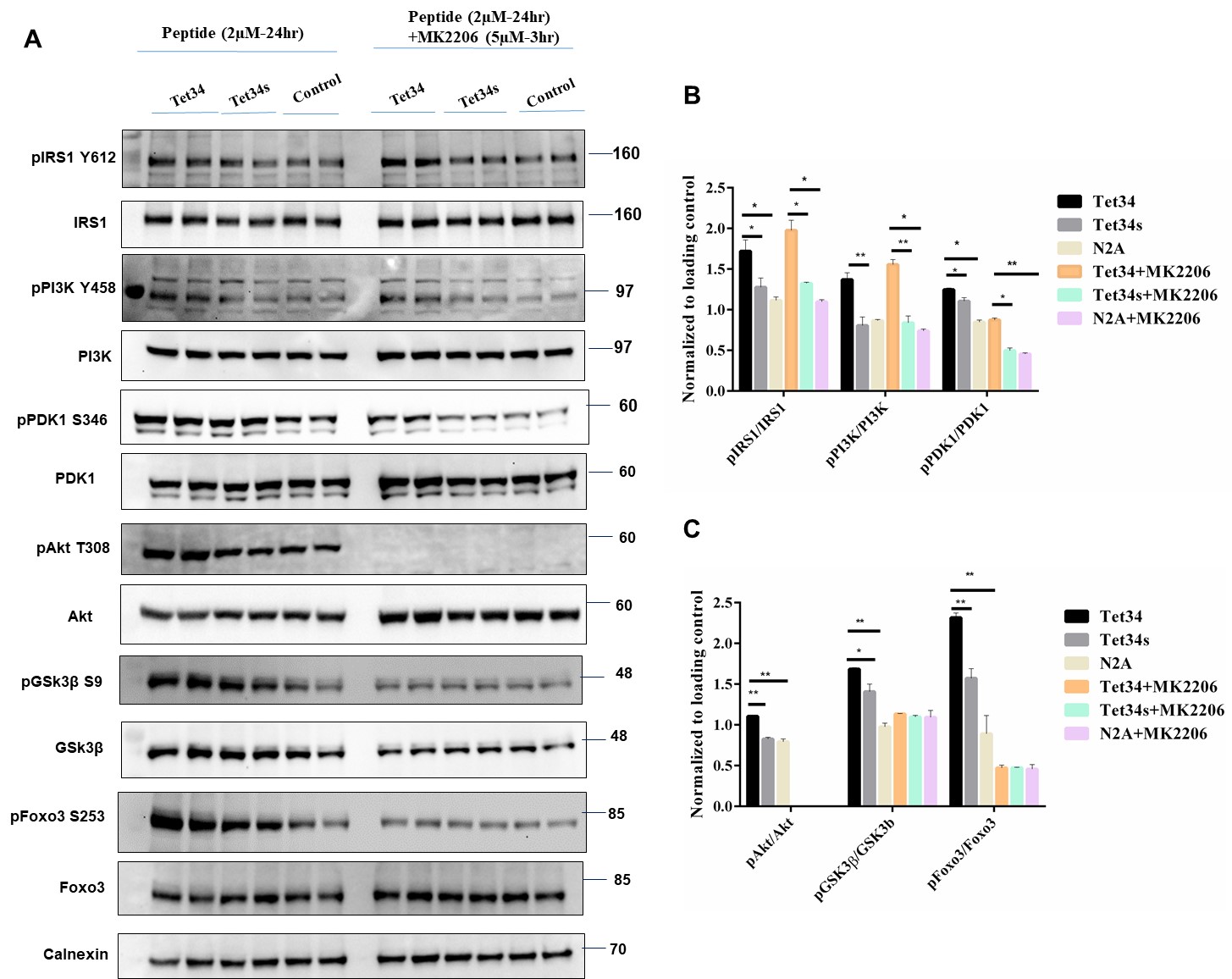
**

**Supplemental Figure S3: CX3CL1-ICD peptides attenuates Foxo activity via IGF1-Rβ/Akt pathway.** (**A**). Western blot analyses from N2A cultures treated with Tet-CX3CL1-ICD peptides in presence or absence of Akt inhibitor, MK2206. Treatment with MK2206 abolished the Tet34 induced effects on Foxo3 phosphorylation. (**B, C**) Bar graphs show protein expression levels normalized to the loading control calnexin. N=3 independent experiments (*P<0.05; **P<0.01; one way ANOVA).


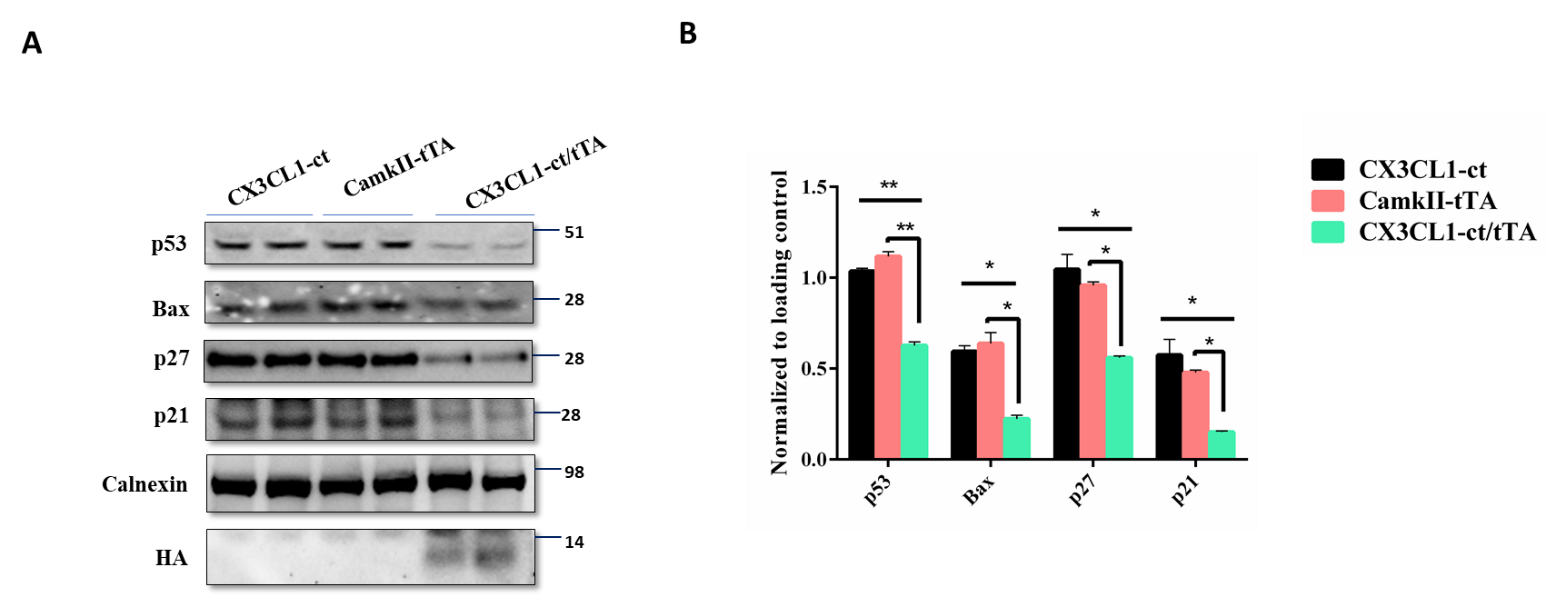


**Supplemental Figure S4: Overexpressed neuronal CX3CL1-ct reduces the pro-apoptotic genes.** (**A**). Western blot analyses from hippocampal lysates of CX3CL1-ct/tTa mice showed significantly down-regulated expression of p53, Bax, p27 and p21. (**B**) Bar graphs show protein expression levels normalized to the loading control calnexin. N=3 independent experiments (*P<0.05; **P<0.01; one way ANOVA).

**
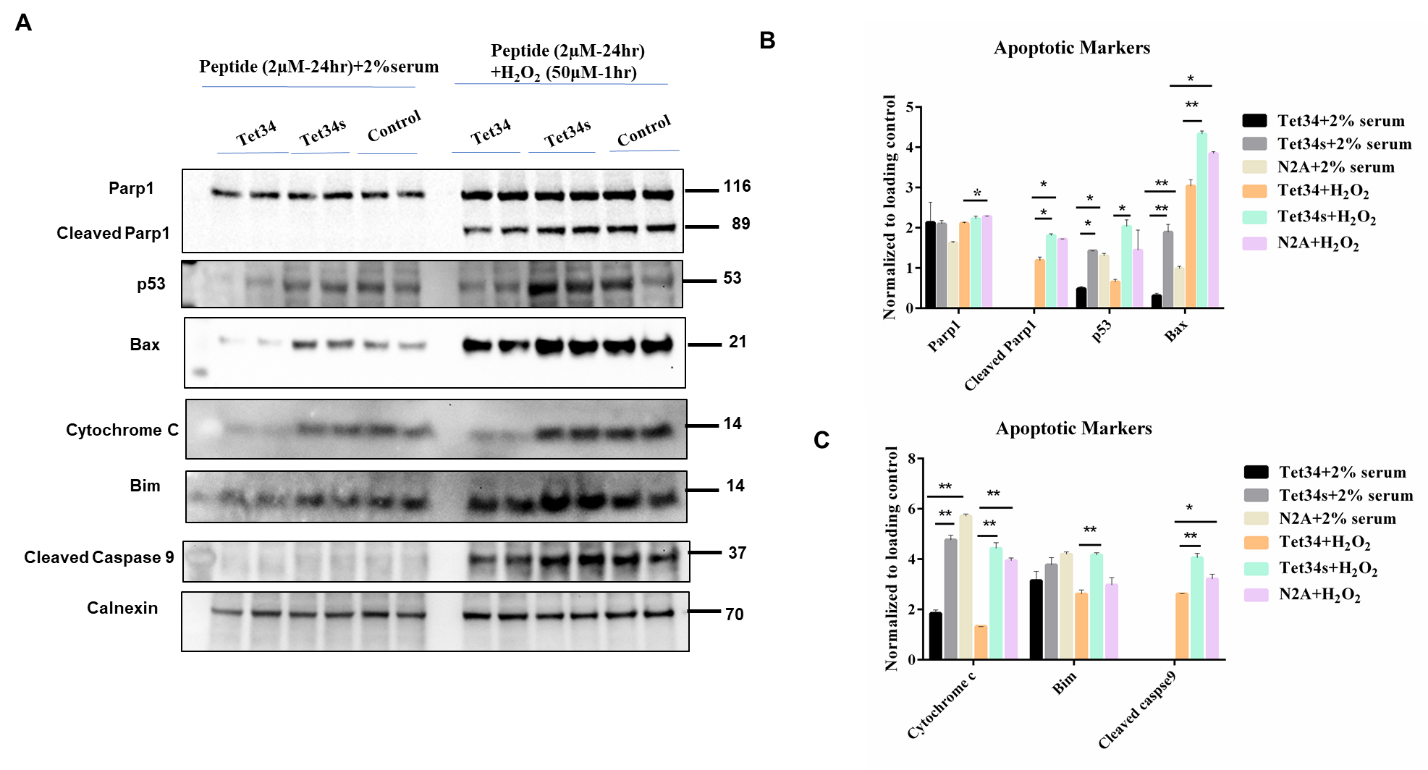
**

**Supplemental Figure S5: CX3CL1-ICD peptides attenuates apoptosis induced by Hydrogen peroxide.** (**A**). Western blot analyses from N2A cultures treated with Tet34 or Tet34s peptides in presence either 2% serum or hydrogen peroxide. Tet34 attenuated the expression of apoptotic markers activated by hydrogen peroxide. (**B, C**) Bar graphs show protein expression levels normalized to the loading control calnexin. N=3 independent experiments (*P<0.05; **P<0.01; one way ANOVA).


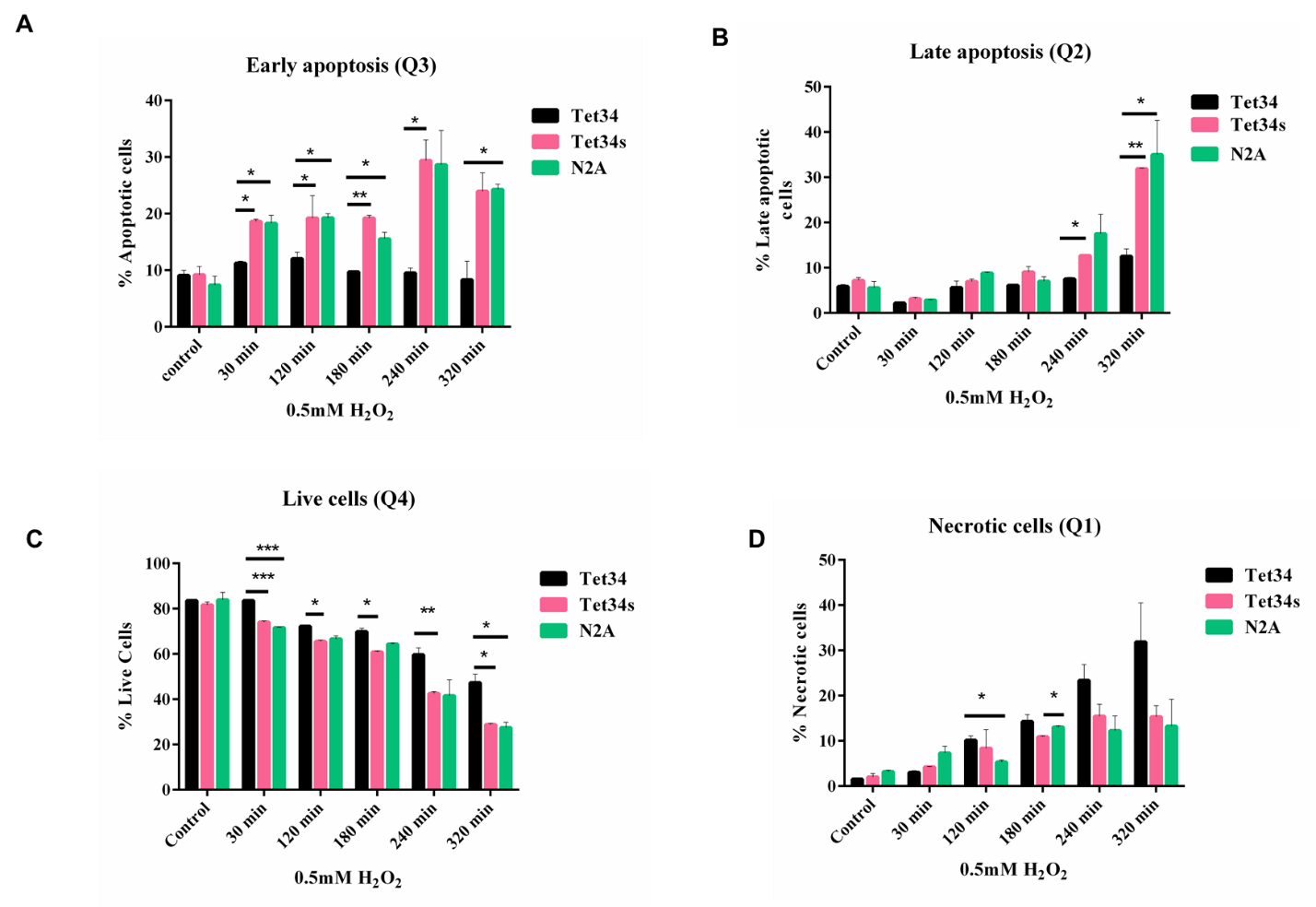


**Supplemental Figure S6: CX3CL1-ICD peptides attenuates apoptosis induced by Hydrogen peroxide.** N2A cells were pretreated with Tet34 or Tet34s peptides for 24hrs followed by hydrogen peroxide treatment. Apoptotic and live cell population was analyzed at different time points by flow cyto metry.N=3 independent experiments (*P<0.05; **P<0.01; Student’s t test).


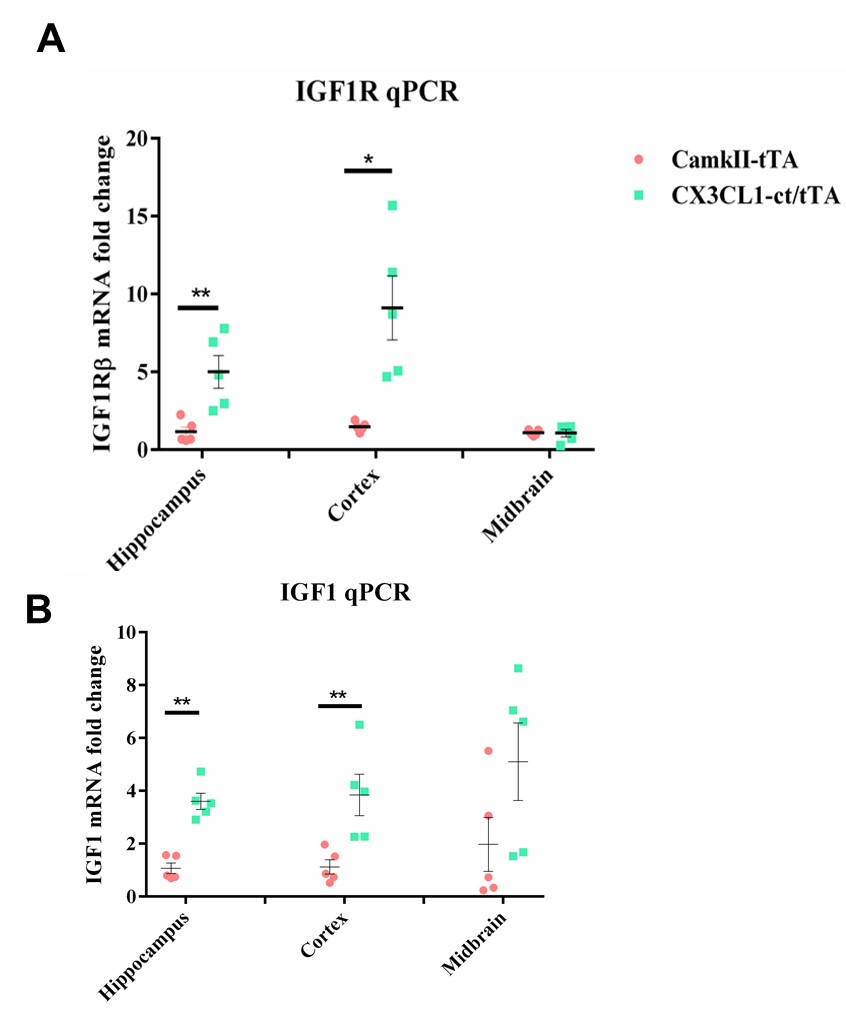


**Supplemental Figure S7: Neuronal overexpression of CX3CL1-ct upregulates IGF-1Rβ mRNA in hippocampus of transgenic mouse.** Messenger RNA was isolated from different regions of the brain and used for real time PCR quantification of IGF-1Rβ (A) and IGF1 (B) mRNA. N=4-5 animals (*P<0.05; **P<0.01; Student’s t test).
